# Optimizing Teamwork in the Operating Room: A Scoping Review of Actionable Teamwork Strategies

**DOI:** 10.7759/cureus.60522

**Published:** 2024-05-17

**Authors:** Nibras Ghanmi, Mostafa Bondok, Cole Etherington, Youssef Saddiki, Isabelle Lefebvre, Pauline Berthelot, Pierre-Marc Dion, Benjamin Raymond, Jeanne Seguin, Pooyan Sekhavati, Sindeed Islam, Sylvain Boet

**Affiliations:** 1 Faculty of Medicine, University of Ottawa, Ottawa, CAN; 2 Department of Anesthesiology, University of British Columbia, Faculty of Medicine, Vancouver, CAN; 3 Clinical Epidemiology Program, Ottawa Hospital Research Institute, Ottawa, CAN; 4 Department of Anesthesiology and Pain Medicine, The Ottawa Hospital, The University of Ottawa, Ottawa, CAN

**Keywords:** interdisciplinary teamwork, scoping review, operating room, patient outcomes, human factors, medical education, healthcare simulation, surgical safety checklist

## Abstract

Suboptimal teamwork in the operating room (OR) is a contributing factor in a significant proportion of preventable complications for surgical patients. Specifying behaviour is fundamental to closing evidence-practice gaps in healthcare. Current teamwork interventions, however, have yet to be synthesized in this way. This scoping review aimed to identify actionable strategies for use during surgery by mapping the existing literature according to the Action, Actor, Context, Target, Time (AACTT) framework. The databases MEDLINE (Medical Literature Analysis and Retrieval System Online), Embase, Cumulated Index to Nursing and Allied Health Literature (CINAHL), Education Resources Information Center (ERIC), Cochrane, Scopus, and PsycINFO were searched from inception to April 5, 2022. Screening and data extraction were conducted in duplicate by pairs of independent reviewers. The search identified 9,289 references after the removal of duplicates. Across 249 studies deemed eligible for inclusion, eight types of teamwork interventions could be mapped according to the AACTT framework: bundle/checklists, protocols, audit and feedback, clinical practice guidelines, environmental change, cognitive aid, education, and other), yet many were ambiguous regarding the actors and actions involved. The 101 included protocol interventions appeared to be among the most actionable for the OR based on the clear specification of ACCTT elements, and their effectiveness should be evaluated and compared in future work.

## Introduction and background

Suboptimal teamwork in the operating room (OR) is a contributing factor in a significant proportion of preventable complications for surgical patients [1-6]. Despite its critical implications for patient safety, best practices for effective teamwork in the OR have yet to be identified [7,8]. Teamwork is defined as the collaborative effort and the dynamic interactions within a group to achieve a common goal. Whether due to poor communication or unclear roles, suboptimal teamwork causes inefficient collaboration, leading to poor performance and increased errors. When such inefficiencies are overcome, effective teamwork is achieved. This can be brought about using teamwork interventions, which focus on improving interactions to bolster performance, safety, and efficiency. Teamwork interventions in the literature include checklists (e.g., Surgical Safety Checklists (SSC)), time-outs or team huddles, tools to facilitate concise communication (e.g., Situation-Background- Assessment-Recommendation (SBAR)), teamwork tools and frameworks (e.g., Team Strategies and Tools to Enhance Performance and Patient Safety (TeamSTEPPS™)), and high-fidelity simulation training or courses. Interventions aiming to improve OR teamwork have yielded mixed results [9-11]. While a lack of clarity regarding actionable teamwork practices is one plausible contributing factor, study design and confounding variables likely also play a role [9,12-16]. Unlike other high-risk industries such as aviation, recommendations about teamwork for the OR continue to revolve around general principles such as "mutual trust" or "adaptability" [17]. Without precise specification of who needs to do what differently, when, where, and how, the development of a shared mental model is challenging at best [18].

Teamwork concepts are typically covered to varying degrees in education and training sessions [10], but less attention is given to well-described and actionable behaviours that can facilitate the identification of best teamwork practices in everyday clinical practice. Previous studies have identified one of the most frequent barriers to effective teamwork within healthcare settings as being the clinicians' lack of knowledge of established best practices or strategies [19,20]. It is thus crucial to empirically establish best practices and disseminate them to clinicians to enhance patient safety. This is particularly significant, given the lack of substantial annual reduction in patient safety events in recent years [21,22].

Specifying expected behaviour is fundamental to closing evidence-practice gaps in healthcare [23-25]. In implementation science, the Action, Actor, Context, Target, Time (AACTT) is an established framework that specifies the necessary elements for an intervention to be considered actionable, thus enhancing intervention effectiveness [26]. Existing systematic reviews have broadly included all forms of teamwork interventions without clearly delineating which interventions contain sufficient behavioural detail for application in the OR [10,11,27]. Identifying actionable teamwork strategies for the OR, rather than broad interventions that emphasize abstract concepts in a classroom setting, is an important step towards providing clinicians with a common ground from which to approach interprofessional teamwork.

This scoping review aims to evaluate the extent to which the current teamwork literature describes actionable practices for use in surgery. We aim to achieve this by mapping studies according to the AACTT framework to identify actionable surgical teamwork practices. This may inform future efforts to improve interprofessional teamwork in the OR.

## Review

Methods

We carried out the scoping review following the updated Preferred Reported Items for Systematic Reviews and Meta-Analyses extension for Scoping Reviews (PRISMA-ScR) guidelines [28]. These guidelines help ensure that scoping reviews possess greater transparency and reliability [28]. Unlike systematic reviews, scoping reviews aim to provide an overview of the available evidence rather than "a summary answer to a discrete research question" [29]. Scoping reviews are useful for answering complex questions in broad areas of literature that have yet to be comprehensively summarized [29,30]. As such, scoping reviews are often preliminary steps to conducting one or several systematic reviews, as the identification of key knowledge gaps informs specific research questions. Since scoping reviews usually contain an expansive purview of information, meta-analytic methods are most often impossible, and risk of bias assessments are not considered essential [29,30].

Information Sources and Search Strategy

Literature searches were conducted using MEDLINE (Medical Literature Analysis and Retrieval System Online), Embase, Cumulated Index to Nursing and Allied Health Literature (CINAHL), Education Resources Information Center (ERIC), Cochrane, Scopus, and PsycINFO databases from inception to April 5, 2022 (See Appendices). The electronic search strategy was developed by an information specialist (AD) in collaboration with the research team and then peer-reviewed in accordance with the Peer Review of Electronic Search Strategies (PRESS) guidelines [31]. A manual screening of the reference lists of included studies was conducted by senior investigators to identify additional potentially relevant articles. All identified articles were imported into DistillerSR (Evidence Partners, Ottawa, Canada), a web-based review software. Duplicate records were removed.

Eligibility Criteria

We defined teamwork interventions as interventions that focus on improving interactions to bolster performance, safety, and efficiency. All empirical study designs were eligible for inclusion provided they explored a teamwork intervention that is actionable, as per the AACTT framework, and could be implemented during the intraoperative period. This meant that an intervention was eligible if it did not require resources/equipment that were not accessible in the OR, did not significantly disrupt OR flow, and did not put at risk patient or staff safety. Studies had to include two or more healthcare professions and could be conducted in any healthcare environment to broaden the possibilities of interventions. Clinical and simulation studies were also eligible for inclusion. Measures of intervention efficacy were not a requirement for inclusion in this scoping review, as our goal was to identify actionable intraoperative teamwork practices or strategies. The elements of the AACTT framework include (I) Action (i.e., behaviour that can be observed and measured), (ii) Actor (i.e., the individual that is doing or could do the behaviour), (iii) Context (i.e., the setting in which the action is performed), (iv) Time (i.e., when the behaviour is performed), and (v) Target (i.e., the person/people with/for whom the action is performed) [26]. Initial eligible study settings included healthcare and other high-risk industries (aviation, military). The protocol was later amended to include only studies conducted in a healthcare setting to provide a more focused review. Only peer-reviewed studies published in English and French were included, while studies in other languages were excluded due to limited resources. Commentaries, editorials, and letters to the editor were not eligible for inclusion.

Screening

Screening was conducted by investigators in two stages using the inclusion and exclusion criteria: (I) title and abstract and (ii) full-length screening. All screeners had a background in research and/or medicine. To ensure standardization, the screening protocol was discussed during an introductory meeting, and a pilot screen was conducted until standardization was achieved.

Two independent investigators screened articles at the title and abstract stage, as well as the full-text stage in duplicate. Excluded studies were flagged with a reason for exclusion and reviewed by two additional healthcare and teamwork experts to confirm the reason for exclusion. Both stages followed the same process, whereby all articles were reviewed in duplicate by two independent reviewers. If consensus could not be achieved, a third reviewer was involved in resolving conflicts.

Data Extraction

A data extraction form was created prior to the literature search and piloted by the research team. There was a training period to trial the form and ensure all reviewers understood the items and documented pilot articles in a unified manner. Data items were extracted using the data extraction form by pairs of independent reviewers. The second reviewer of each pair verified the data extraction of the first reviewer for accuracy, and any disagreements between the two reviewers were flagged for discussion. If consensus could not be reached, a third reviewer was involved. When data items were inadequately reported in the full text, attempts at contacting the original authors were made to clarify and confirm relevant details.

Data Items and Synthesis of Results

The data collected included publication details (e.g., first author, journal, year of publication, country of origin), study design, sample and participants, setting, title, type, description of the teamwork intervention, and which of the AACTT elements the intervention specified. Data collection and synthesis were conducted by one reviewer, with a second reviewer verifying the accuracy and consistency of the extracted data and classifications. Data extracted from the included studies were organized into distinct domains based on established categories of practice-changing interventions. These domains encompassed various types of intraoperative teamwork interventions, including bundle/checklists, protocols, audit and feedback, clinical practice guidelines, environmental changes, cognitive aids, education, and others [32]. Each intervention type was systematically identified and categorized to facilitate comprehensive analysis and synthesis of the literature. Data were organized according to the AACTT framework, which served as a guiding framework for mapping out the specific elements of each intervention, including the actions undertaken, the actors involved, the contextual factors influencing implementation, the intended targets or recipients of the intervention, and the temporal aspects of intervention delivery. This categorization facilitated a nuanced understanding of the teamwork interventions' characteristics and actionability in the intraoperative period.

Quantitative and qualitative data extracted from included studies were subject to comprehensive analysis to elucidate key findings and insights. Quantitative data, such as the frequency of intervention types across studies, were analyzed using appropriate summary statistics, including counts and percentages. This quantitative analysis provided a quantitative overview of the prevalence and distribution of different intervention types within the literature. Qualitative data, including descriptions of intervention components and their associated AACTT elements, were subjected to thematic analysis. The thematic analysis involved the identification of recurrent themes, patterns, and trends within the extracted data. By systematically examining the qualitative data, commonalities, variations, and nuances in intervention characteristics and implementation strategies were identified. These thematic insights provided a qualitative understanding of the diverse approaches to intraoperative teamwork interventions and illuminated the contextual factors shaping their implementation and effectiveness.

Results

Study Selection

**Figure 1:**
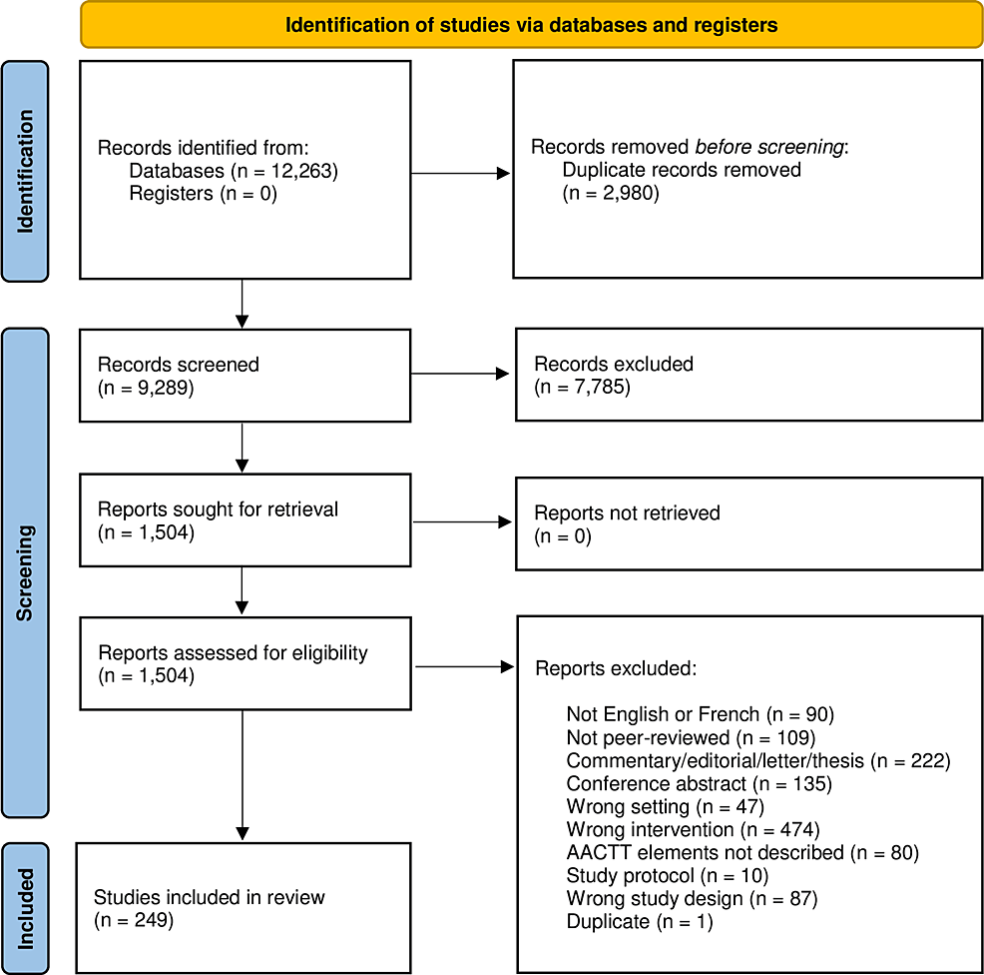
PRISMA Flowchart PRISMA: Preferred Reporting Items for Systematic Reviews and Meta-Analyses

There were 9,289 relevant studies identified from our literature search. Of these, 7,785 were excluded at the title and abstract screening, and 1,255 were excluded at full-text screening. This resulted in a total of 249 articles included in this review (Figure 1).

Summary of Study Characteristics

Of the 249 included studies, 137 (55.0%) were published in the United States. A wide range of study designs were observed, with the most common being before-and-after studies (n=88; 35.3%) and non-randomized experimental studies (n=66; 26.5%). More than two-thirds of studies (n=172; 69.1%) involved the OR and corresponding healthcare professionals such as surgeons, anesthesiologists, and circulating or scrub nurses. Sample populations (i.e., the number of each type of professional involved) were not consistently reported across studies. Outside of the OR, study settings included hospital wards (n=17; 6.8%), obstetrics (n=6; 2.4%), post-anesthesia or intensive care units (n=8; 3.2%), trauma rooms (n=10; 4%), emergency departments (n=5; 2%), multiple health care settings (n=23; 9.2%), and other settings such as long-term care and outpatient clinics (n=8; 3.2%). Education was the most frequently reported type of intervention (n=108; 43.4%), while bundle/checklists were the second most reported (n=76; 30.5%). Other types of interventions described across included studies were protocols (n=38; 15.3%), audit and feedback (n=16; 6.4%), clinical practice guidelines (n=1; <1%), environment improvement (n=1, <1%), cognitive aids (n=5; 2%), and other interventions such as hands-free communication devices (n=4; 1.6%).

AACTT Specifications Across Included Studies

A summary of the AACTT specifications across the included studies is provided in Table 1. The action (i.e., behaviour) specified by most studies (n=133; 53.4%) was to follow a series of steps or tasks listed in the intervention bundle or checklist; however, only 76 (30.5%) of studies specified the actor (i.e., the person who performs the action). For example, the Surgical Patient Safety System (SURPASS) checklist described by de Vries et al. [33] specifies which individual team member (e.g., anesthesiologist) is responsible for completing each item (e.g., checking patient allergies and equipment) across each phase in the surgical pathway (e.g., at the OR time-out). The SSC was described by 24 studies (9.6%) and specifies many individual actions occurring throughout the procedure but did not always precisely specify the actor or target. For example, the checklist states "with at least nurse and anesthetist" regarding the actions that are to take place before induction of anesthesia but does not explicitly state who does which item. Similarly, actions before skin incision and before the patient leaves the OR are stated to take place "with the nurse, anesthetist, and surgeon," but the specific actor and target of several checklist items are not systematically indicated.

**Table 1:** Summary of Action, Actor, Context, Target, Time (AACTT) framework across included studies %: Represents the number of studies over the total number of studies, N, where N=249.

Framework Component	Number of studies (%)
Action	
Follow a series of steps or tasks listed in a checklist or protocol	133 (53.4%)
Take specific individual action (e.g., silence mobile devices)	101 (40.5%)
Use specified electronic tool (e.g., hands free communication device)	15 (6%)
Actor	
Any/all team members	158 (63.4%)
Individual (staff) team member as specified	76 (30.5%)
Patient & team members as specified	3 (1.2%)
Trainee	11 (4.4%)
Any team member except trainees and travel nurses	1 (<1%)
Context	
Hospital ward	17 (6.8%)
Operating room	172 (69.1%)
Obstetrics	6 (2.4%)
Post-anesthesia care unit	1 (<1%)
Intensive care unit	7 (2.8%)
Trauma centre	10 (4%)
Multiple health care settings	23 (9.2%)
Emergency department	5 (2.0%)
Other (e.g., long-term care, outpatient clinic)	8 (3.2%)
Target	
Any/all team members	161 (64.7%)
Individual (staff) team member as specified	40 (16.1%)
Patient	6 (2.4%)
Patient and team members as specified	35 (14.1%)
Trainee	7 (2.8%)
Time	
At specified intervals	18 (7.2%)
As needed	26 (10.4%)
Critical situation	39 (15.6%)
Handover	13 (5.2%)
Before procedure	34 (13.6%)
Before procedure and as needed	1 (<1%)
During procedure	51 (20.4%)
Before and after procedure	11 (4.4%)
Before, during and after procedure	45 (18%)
Not reported	3 (1%)

Interventions involving specific individual actions rather than a multi-step, multi-actor bundle or checklist were described by 101 studies (40.5%). A representative example of 10 studies is shown in Table 2. One example is closed-loop communication [34], which involves the components of a callout (i.e., verbal order), check back (i.e., confirmation that information was received), and closing the loop (i.e., the acknowledgement that the receiver correctly understood the information). Other examples involved protocols to minimize distractions. The noise reduction intervention described by Wright et al., for example, requires OR team members to eliminate non-essential conversation, turn the volume down or off on electronics, silence mobile devices, and avoid the use of unnecessary instruments or devices that increase noise levels (Table 2) [35]. Similarly, the "sterile cockpit" protocol introduced by West et al. aiming to improve the efficacy and safety of nursing assistants (NAs) specifies the actions for registered nurses to take, such as "engage the NAs only in professional conversations" and "take phone calls and messages for the NAs" (Table 2) [36].

**Table 2:** Examples of specific teamwork behaviours identified across relevant studies (N=10) OR: operating room; NA: nursing assistant

Study	Action	Actor	Context	Target	Time
Sucharew and Macaluso, 2019 [29]	Use closed-loop communication: Callout – verbal order Check back – confirm information received Closing the loop – acknowledge correct understanding of information	Team leader	Trauma centre	Any team member	During procedure
Sinuff et al., 2013 [32]	Classify situation urgency with the Traffic Lights tool: Red alert – life-threatening emergency Amber assist – help is required within minutes Green query – advice/non-urgent assistance required	Anesthesia trainee	Operating room	Staff anesthesiologist	During procedure
Pham et al., 2014 [30]	Reduce noise by eliminating non-essential conversation: Turn the volume down or off on electronics, slience mobile devices, avoid the use of instruments or devices that increase noise levels if they are unnecessary at that time	All team members	OR	All team members	During anesthesia induction, surgical briefing, specimen collection, final surgical counts and debriefing, and anesthesia emergence
McGowan et al., 2015 [31]	Minimize distractions and interruptions: Intercept individuals who would otherwise have contact NAs, take phone calls and messages for the NAs, answer call lights and patient requests that normally would have been handled by the NAs, engage the NAs only in professional conversations, restrict overhead paging (use phones or nurse pagers only)	Registered Nurses	Cardiac medicine unit	NAs	Any time during the shift
De Vries et al., 2011 [33]	Remind the team when OR traffic is excessive.	Surgeon	OR	All team members	During procedure
El-Shafy et al., 2018 [34]	Follow the established communication structure: (Surgeon) Call out the colour for the next tool (Surgeon) Say “disabled” once the tool is disabled on the control panel (Nurse) Say “ready” once the new tool is secured	Surgeon and Nurse	OR	Surgeon and Nurse	During procedure
Wright, 2016 [35]; West et al., 2012 [36]	Communicate using the SBAR tool. Situation: describe the current status of the patient and provide a concise statement of the problem; Background: provide pertinent and brief information related to the situation; Assessment: provide an overall analysis of the patient and their status; Recommendation: explain what exactly needs to be done after the original team member leaves	Giver of information	OR/intensive care unit/post-anesthetic care unit	Receiver of information	Handover

The context (i.e., setting) of the interventions across the included studies was largely in the OR itself (n=172; 69.1%) with the action specified as taking place during the procedure (n=51; 20.5%), without consistently indicating an exact point in or duration of time. By contrast, other studies indicated a specific time for the action to occur, such as during a critical situation (n=39; 15.7%) or handover (n=13; 5.2%). Interventions tended to target team members in general (n=161; 63.8%), or an individual specified team member (n=40; 16.1%). Patients were included as the target (i.e., the person for whom the action is performed) of the interventions in 6 (2.4%) studies.

Discussion

This scoping review provides an overview of actionable teamwork practices that could be implemented intraoperatively. We identified eight different types of teamwork interventions across 249 studies that included practices or strategies that were actionable, and thus mapped according to the AACTT framework. The included interventions typically involved many unspecific actions and actors. Conversely, a smaller number of included studies reported on protocol interventions with single, well-defined actions required of all or specific team members. Within these interventions, the prescribed actions are related primarily to improving communication practices or reducing distractions.

A potential advantage of the communication and distraction protocol interventions identified in this review is that they contain fewer and more specific behavioural specifications (e.g., closed-loop communication, silence mobile devices). By comparison, the included bundle/checklist interventions are more complex, as they contain many possible answers to the key implementation question "who needs to do what differently" [26]. This review ultimately raises important questions about AACTT specification among widely implemented interventions. It is possible that the lack of actionable descriptions of teamwork interventions may explain the mixed results observed regarding the effectiveness of such teamwork interventions [9-11,37,38]. Although these interventions may initially appear straightforward, the ambiguity of the AACTT elements may undermine their effectiveness. Other studies have confirmed that ambiguity remains one of the key implementation and compliance challenges affecting the SSC [39]. These findings, along with the results of this scoping review, speak to the common implementation challenge of balancing fidelity (i.e., the intervention is delivered, received, and enacted as intended) and adaptation (i.e., adjustments to the original intervention made by implementers or users as they go about delivering an intervention) [40,41]. Fidelity may be easier to accomplish with the identified communication and distraction protocols, in comparison to the bundle/checklist interventions, and adaptation may be less variable. Accordingly, the reproducibility and sustained effectiveness of the interventions may be enhanced.

Implementing specific teamwork practices or strategies also has the potential to establish effective teamwork as a routine practice across intraoperative settings. Indeed, in real-world healthcare settings, interventions that explicitly designate specific roles for actors and/or targets are more likely to be actionable for several reasons. Firstly, such interventions provide clarity and accountability by clearly delineating the responsibilities of each team member, ensuring that everyone understands their role in implementing the intervention. This clarity enhances accountability within the healthcare team and minimizes confusion regarding task ownership. Secondly, interventions with clearly defined roles are more effectively implemented, as they reduce ambiguity and enable healthcare professionals to carry out their tasks accurately and consistently [20]. Thirdly, specifying roles allows for customization and adaptation of interventions to fit the unique needs and dynamics of different healthcare settings, fostering flexibility and scalability. Additionally, clear role assignments facilitate communication and collaboration among team members, promoting seamless coordination of actions and effective achievement of common goals. Finally, interventions with specific roles are easier to evaluate and provide feedback on, enabling continuous quality improvement and optimization of outcomes [42]. Overall, interventions that name specific roles for actors and/or targets enhance clarity, accountability, implementation effectiveness, customization, communication, collaboration, and evaluation, making them more actionable and conducive to successful adoption in real-world healthcare contexts [43]. In addition, clinicians’ limited knowledge of specific practices or strategies for engaging in effective teamwork [19,20] is further indicative of the value of interventions that specify the AACTT elements. Studies demonstrate a shared mental model is an essential characteristic of high-performing teams [44-46]. Therefore, at minimum, the teamwork practices or strategies elicited from these types of interventions could promote a common understanding of effective teamwork among interprofessional team members.

Given the proliferation of checklists over the last decade as a strategy for reducing medical errors [47] and the widespread use of the SSC in particular [48,49], it is not surprising that these were among the most identified interventions. While several reviews have demonstrated at least moderate effectiveness of the SSC in improving patient outcomes, there is less evidence that the checklist consistently enhances teamwork [50,51]. In fact, when used sub-optimally, checklists can even negatively impact team functioning [52]. For example, the checklist can reinforce professional divisions by failing to include all individuals or professional groups during the "checking" process. Many studies also suggest the implementation of and compliance with the SSC remains challenging [53-55], and that the checklist "may encourage box-ticking without true fidelity to (its) communications and process assurance aspects" [56]. Future studies utilizing checklists can be improved by ensuring all checklist items can be mapped to all components of the AACTT framework. Analyzing teamwork interventions through the lens of the ACCTT framework is valuable as it specifies how an intervention should be applied and may therefore facilitate implementation. In cases where checklists have previously failed or were deemed to be ineffective, specific communication interventions, such as those identified in this review, may provide a more direct way of improving teamwork rather than expecting it to be a by-product of various task-related checkboxes.

The implementation of any teamwork intervention should take local barriers and enablers into consideration [18]. It could be expected that specific behavioural interventions are more amenable to local tailoring than those which are more ambiguous, and future research may wish to investigate this hypothesis. Differences in compliance rates between specific versus ambiguous interventions may also be an insightful area of research to pursue.

Strengths and Limitations

This scoping review involved a comprehensive search strategy and a rigorous screening process. Nevertheless, it is likely that some relevant studies were missed based on inconsistencies in reporting across studies and the potential subjectivity of reviewers in determining whether interventions satisfied AACTT criteria. To mitigate this risk, screeners were trained prior to conducting the review; all screening was conducted in duplicate, and exclusion decisions were reviewed and verified by two independent research team members.

Although our review focused on practices that can be conducted inside the clinical OR, we recognize that other types of interventions can still be of value. The strategies we identified may be advantageous in that they can be incorporated into daily clinical practice and provide healthcare professionals with a shared foundation for effective teamwork. This, of course, does not preclude the use of additional interventions targeting individual provider skills, professional hierarchies, or organizational culture. Another limitation of this study is the deductive methodology used to identify actionable practices for use during surgery. This specific method was chosen to ensure that our study was based on a recognized framework with pre-defined categories that provide a basis for practical application. We recognize, however, that this approach may prevent us from identifying new categories of interventions that do not fit within the established categories or fit in a non-specific "other" category. In the future, employing an inductive approach may capture a more comprehensive list of practices that go beyond these pre-defined categories. Finally, we did not assess the quality of the included studies or their effectiveness, as this is typically not required for a scoping review. The goal of this scoping review was to identify actionable teamwork practices for the OR, rather than to assess intervention effectiveness. We intend to conduct a subsequent study using the identified strategies to further explore the most promising strategies from the perspective of the AACTT framework for routine application in the OR. Given its advantages, teamwork interventions should be designed and described with the AACTT framework in mind, which may improve the actionability and duplicability of interventions described in future research. Systematic reviews assessing the effectiveness of specific types of interventions based on study-reported outcomes could be among the next steps. Further studies on the varied implementation process may also help to better understand the conflicting success achieved with various teamwork strategies.

## Conclusions

This scoping review identifies actionable teamwork practices for intraoperative implementation, encompassing eight intervention types across 249 studies by mapping the existing literature according to the AACTT framework. While most interventions lacked specificity in actions and actors, protocol interventions offered clear roles, primarily focusing on communication improvement and distraction reduction. Specific role designation enhances clarity, accountability, and implementation effectiveness. Clear role assignments facilitate communication, collaboration, and evaluation, promoting effective teamwork and shared mental models among interprofessional team members. The implementation of any teamwork intervention should take local barriers and enablers into consideration, and tailor interventions accordingly. Future research may consider evaluating differences in compliance rates between specific versus ambiguous interventions.

## Appendices

**Table 3:** Electronic search strategy

Line Number	Search Terms
Ovid MEDLINE(R) ALL	
1	*patient care team/ or Patient Care Team/st [Standards]
2	team*.ti,kw.
3	teamwork.tw,kw.
4	team member*.tw,kw.
5	(team* adj2 (behaviour or behavior or situation or performance)).tw.
6	or/1-5
7	team* communication.tw,kw.
8	communication.ti,kw.
9	communication strateg*.tw,kw.
10	Communication/
11	Verbal Behavior/
12	Nonverbal Communication/ or ((nonverbal or non verbal) adj3 (communicat* or strateg* or interaction*)).tw.
13	(hand signal* or visual signal*).tw,kw.
14	(team* adj3 training).tw.
15	checklist/ or (checklist* or check list*).tw,kw.
16	(script* or whiteboard* or toolkit*).tw,kw.
17	(prompt or prompts or cue or cues).tw,kw.
18	(sbar or callout* or call out* or checkback* or check back* or DESC).tw,kw.
19	(situation and background and assessment and recommendation).tw.
20	(communication adj3 (closed or loop*)).tw.
21	"clos* the loop".tw,kw.
22	"pass the baton".tw,kw.
23	(crew resource or CRM).tw,kw.
24	"two challenge rule".tw,kw.
25	("speak up" or "speaking up").tw,kw.
26	or/8-25
27	6 and 26
28	aviation/ or Military Personnel/ or Nuclear Power Plants/
29	(aviation or aerospace or aeronautic* or cockpit or military or aviator* or pilots or fighter pilot or flight personnel or battlefield* or power plant*).tw,kw.
30	(high risk adj2 (industr*or environment* or setting*)).tw.
31	exp Surgical Procedures, Operative/
32	Operating Rooms/
33	(operating adj2 (room* or theatre*)).tw,kw.
34	surgical team*.tw,kw.
35	Intraoperative Period/ or ((perioperative or intraoperative) adj2 (period or setting or environment)).tw.
36	((during or undergoing) adj3 surgery).tw.
37	or/28-36
38	7 or 27
39	37 and 38
40	limit 39 to dt=20190615-20220404
Embase	
1	teamwork/
2	team*.ti.
3	teamwork.tw.
4	(team member or team members).tw.
5	(team* adj2 (behaviour or behavior or situation or performance)).tw.
6	or/1-5
7	team* communication.tw.
8	communication.ti.
9	interpersonal communication/
10	verbal behavior/ or verbal communication/
11	nonverbal communication/ or ((nonverbal or non verbal) adj3 (communicat* or strateg* or interaction*)).tw.
12	communication strateg*.tw,kw.
13	(team* adj3 training).tw.
14	checklist/ or checklist*.tw.
15	(script* or whiteboard* or toolkit*).tw.
16	(prompt or prompts or cue or cues).tw.
17	(visual signal* or hand signal*).tw.
18	(sbar or callout* or call out* or checkback* or check back* or DESC).tw.
19	(situation and background and assessment and recommendation).tw.
20	(communication adj3 (closed or loop*)).tw.
21	"clos* the loop".tw.
22	"pass the baton".tw.
23	"two challenge rule".tw.
24	("speak up" or "speaking up").tw.
25	crew resource.tw.
26	CRM.tw.
27	or/8-26
28	6 and 27
29	7 or 28
30	aviation/
31	airplane crew/
32	nuclear power plant/
33	(aviation or aerospace or aeronautic* or cockpit or military or aviator* or flight personnel or pilots or fighter pilot* or battlefield* or power plant*).tw.
34	aerospace medicine/
35	(high risk adj2 (industr*or environment* or setting*)).tw.
36	exp *surgery/
37	operating room/
38	(operating adj2 (room* or theatre*)).tw.
39	operating room personnel/
40	intraoperative period/ or ((perioperative or intraoperative) adj2 (period or setting or environment)).tw.
41	surgical team.tw.
42	or/30-41
43	29 and 42
44	limit 43 to dc=20190615-20220404
APA PsycInfo	
1	Teams/ or Work Teams/
2	team*.ti.
3	teamwork.tw.
4	team member*.tw.
5	(team* adj2 (behaviour or behavior or situation or performance)).tw.
6	or/1-5
7	team* communication*.tw.
8	communicat*.ti.
9	Interpersonal Communication/ or Interpersonal Interaction/
10	exp nonverbal communication/
11	((nonverbal or non verbal) adj3 (communicat* or interaction*)).tw.
12	(visual signal* or hand signal*).tw.
13	oral communication/
14	communication strateg*.tw.
15	(team* adj3 training).tw.
16	"CHECKLIST (TESTING)"/
17	(checklist* or check list*).tw.
18	(script* or whiteboard* or toolkit*).tw.
19	CUES/
20	(prompt or prompts or cue or cues).tw.
21	(sbar or callout* or call out* or checkback* or check back* or DESC).tw.
22	(situation and background and assessment and recommendation).tw.
23	(communication adj3 (closed or loop*)).tw.
24	"clos* the loop".tw.
25	"pass the baton".tw.
26	crew resource.tw.
27	"two challenge rule".tw.
28	("speak up" or "speaking up").tw.
29	or/8-28
30	6 and 29
31	7 or 30
32	AVIATION SAFETY/ or AVIATION/
33	Aircraft Pilots/ or Air Force Personnel/ or Military Personnel/ or Aerospace Personnel/
34	(aviation or aerospace or aeronautic* or cockpit or military or aviator* or flight personnel or pilots or fighter pilot* or battlefield* or power plant*).tw.
35	(high risk adj2 (industr*or environment* or setting*)).tw.
36	Surgery/
37	(operating adj2 (room* or theatre*)).tw.
38	surgical team*.tw.
39	((during or undergoing) adj3 surgery).tw.
40	(perioperative or intraoperative).tw.
41	or/32-40
42	31 and 41
43	limit 42 to "0200 book"
44	42 not 43
45	limit 44 to up=20190615-20220404
ERIC	
1	Teamwork/
2	teamwork.tw.
3	team member*.tw.
4	team*.ti.
5	(team* adj2 (behaviour or behavior or situation or performance)).tw.
6	or/1-5
7	team* communication.tw.
8	"Communication (Thought Transfer)"/ or Communication Strategies/
9	communication strateg*.tw.
10	team training/ or (team* adj3 training).tw.
11	Check Lists/
12	checklist*.tw.
13	(script* or whiteboard* or toolkit*).tw.
14	Scripts/
15	(prompt or prompts).tw.
16	Cues/ or (cue or cues).tw.
17	(sbar or callout* or call out* or checkback* or check back* or DESC).tw.
18	Active Learning/
19	(situation and background and assessment and recommendation).tw.
20	(communication adj3 (closed or loop*)).tw.
21	"clos* the loop".tw.
22	"pass the baton".tw.
23	crew resource.tw.
24	"two challenge rule".tw.
25	("speak up" or "speaking up").tw.
26	CRM.tw.
27	Nonverbal Communication/ or Verbal Communication/
28	((nonverbal or non verbal) adj3 (communicat* or interaction*)).tw.
29	or/8-28
30	6 and 29
31	7 or 30
32	Flight Training/
33	(aviation or aerospace or aeronautic* or cockpit or military or aviator* or pilots of fighter pilot* or flight personnel or battlefield* or power plant*).tw.
34	Military Personnel/ or Armed Forces/
35	(high risk adj2 (industr*or environment* or setting*)).tw.
36	(operating adj2 (room* or theatre*)).tw.
37	Surgery/
38	surgical team*.tw.
39	(perioperative or intraoperative).tw.
40	((during or undergoing) adj3 surgery).tw.
41	or/32-40
42	31 and 41
43	limit 42 to 04012022
CINAHL	
S1	(MH "Teamwork")
S2	TI teamwork or team member* OR AB teamwork or team member*
S3	TI team*
S4	TI ( (team* N2 (behaviour or behavior or situation or performance)) ) OR AB ( (team* N2 (behaviour or behavior or situation or performance)) )
S5	S1 OR S2 OR S3 OR S4
S6	(MH "Communication") OR (MH "Nonverbal Communication+") OR (MH "Verbal Behavior+")
S7	TI communication or ((nonverbal or non verbal) N3 (communicat* or interaction*)) OR AB communication strateg* or ((nonverbal or non verbal) N3 (communicat* or interaction*))
S8	TI checklist* or ( (hand signal* or visual signal*) ) OR AB checklist* or ( (hand signal* or visual signal*) ) OR SU checklist*
S9	TI ( (script* or whiteboard* or toolkit* or prompt or prompts or cue or cues) ) OR AB ( (script* or whiteboard* or toolkit* or prompt or prompts or cue or cues) )
S10	TI ( (crew resource) ) OR AB ( (crew resource) )
S11	TI ( (sbar or callout* or call out* or checkback* or check back* or DESC) ) OR AB ( (sbar or callout* or call out* or checkback* or check back* or DESC) )
S12	TI ( (situation and background and assessment and recommendation) ) OR AB ( (situation and background and assessment and recommendation) )
S13	TI ( (communication N3 (closed or loop*)) ) OR AB ( (communication N3 (closed or loop*)) )
S14	TI "pass the baton" OR AB "pass the baton"
S15	TI "two challenge rule" OR AB "two challenge rule"
S16	TI ( ("speak up" or "speaking up") ) OR AB ( ("speak up" or "speaking up") )
S17	TI "clos* the loop" OR AB "clos* the loop"
S18	S6 OR S7 OR S8 OR S9 OR S10 OR S11 OR S12 OR S13 OR S14 OR S15 OR S16 OR S17
S19	TI ( (aviation or aerospace or aeronautic* or cockpit or military or aviator* or pilots or fighter pilot or flight personnel or battlefield* or power plant*) ) OR AB ( (aviation or aerospace or aeronautic* or cockpit or military or aviator* or pilots or fighter pilot or flight personnel or battlefield* or power plant*) )
S20	(MH "Aviation+")
S21	SU military
S22	TI ( (operating N2 (room* or theatre*)) ) OR AB ( (operating N2 (room* or theatre*)) )
S23	SU operating rooms OR (MH "Intraoperative Period")
S24	TI ( ((perioperative or intraoperative) N2 (period or setting or environment)) ) OR AB ( ((perioperative or intraoperative) N2 (period or setting or environment)) )
S25	TI surgical team* OR AB surgical team*
S26	TI ( ((during or undergoing) N3 surgery) ) OR AB ( ((during or undergoing) N3 surgery) )
S27	(MH "Surgery, Operative+")
S28	TI ( (high risk N2 (industr*or environment* or setting*)) ) OR AB ( (high risk N2 (industr*or environment* or setting*)) )
S29	S19 OR S20 OR S21 OR S22 OR S23 OR S24 OR S25 OR S26 OR S27 OR S28
S30	TI team* communication OR AB team* communication
S31	S5 AND S18
S32	S30 OR S31
S33	S29 AND S32
Cochrane Central Register of Controlled Trials	
1	..nlpx "query=MeSH descriptor: [Patient Care Team] this term only","desiredResults=10000","minHitsDivisor=7","permitHyponyms=NO","lowestVocabularySearchLevel=none","phrasesBroken=NO","speedWanted=NoHypos","comment=Including Related Terms","elimEnable=NO","constraintMinTerms=2"
2	..nlpx "query=(team*):ti","desiredResults=10000","minHitsDivisor=7","permitHyponyms=NO","lowestVocabularySearchLevel=none","phrasesBroken=NO","speedWanted=NoHypos","comment=Including Related Terms","elimEnable=NO","constraintMinTerms=2"
3	..nlpx "query=(teamwork):ti,ab,kw","desiredResults=10000","minHitsDivisor=7","permitHyponyms=NO","lowestVocabularySearchLevel=none","phrasesBroken=NO","speedWanted=NoHypos","comment=Including Related Terms","elimEnable=NO","constraintMinTerms=2"
4	..nlpx "query=(team* NEAR/2 (behaviour or behavior or situation or performance))","desiredResults=10000","minHitsDivisor=7","permitHyponyms=NO","lowestVocabularySearchLevel=none","phrasesBroken=NO","speedWanted=NoHypos","comment=Including Related Terms","elimEnable=NO","constraintMinTerms=2"
5	..nlpx "query=("team member" or "team members"):ti,ab,kw","desiredResults=10000","minHitsDivisor=7","permitHyponyms=NO","lowestVocabularySearchLevel=none","phrasesBroken=NO","speedWanted=NoHypos","comment=Including Related Terms","elimEnable=NO","constraintMinTerms=2"
6	1 or 2 or 3 or 4 or 5
7	..nlpx "query=(team* communication):ti,ab,kw","desiredResults=10000","minHitsDivisor=7","permitHyponyms=NO","lowestVocabularySearchLevel=none","phrasesBroken=NO","speedWanted=NoHypos","comment=Including Related Terms","elimEnable=NO","constraintMinTerms=2"
8	..nlpx "query=MeSH descriptor: [Communication] explode all trees","desiredResults=10000","minHitsDivisor=7","permitHyponyms=NO","lowestVocabularySearchLevel=none","phrasesBroken=NO","speedWanted=NoHypos","comment=Including Related Terms","elimEnable=NO","constraintMinTerms=2"
9	..nlpx "query=((nonverbal or non verbal) NEAR/3 (communicat* or interaction*)):ti,ab,kw","desiredResults=10000","minHitsDivisor=7","permitHyponyms=NO","lowestVocabularySearchLevel=none","phrasesBroken=NO","speedWanted=NoHypos","comment=Including Related Terms","elimEnable=NO","constraintMinTerms=2"
10	..nlpx "query=(communication strateg*):ti,ab,kw","desiredResults=10000","minHitsDivisor=7","permitHyponyms=NO","lowestVocabularySearchLevel=none","phrasesBroken=NO","speedWanted=NoHypos","comment=Including Related Terms","elimEnable=NO","constraintMinTerms=2"
11	..nlpx "query=((team* NEAR/3 training)):ti,ab,kw","desiredResults=10000","minHitsDivisor=7","permitHyponyms=NO","lowestVocabularySearchLevel=none","phrasesBroken=NO","speedWanted=NoHypos","comment=Including Related Terms","elimEnable=NO","constraintMinTerms=2"
12	..nlpx "query=MeSH descriptor: [Checklist] explode all trees","desiredResults=10000","minHitsDivisor=7","permitHyponyms=NO","lowestVocabularySearchLevel=none","phrasesBroken=NO","speedWanted=NoHypos","comment=Including Related Terms","elimEnable=NO","constraintMinTerms=2"
13	..nlpx "query=(checklist*):ti,ab,kw","desiredResults=10000","minHitsDivisor=7","permitHyponyms=NO","lowestVocabularySearchLevel=none","phrasesBroken=NO","speedWanted=NoHypos","comment=Including Related Terms","elimEnable=NO","constraintMinTerms=2"
14	..nlpx "query=((script* or whiteboard* or toolkit*)):ti,ab,kw","desiredResults=10000","minHitsDivisor=7","permitHyponyms=NO","lowestVocabularySearchLevel=none","phrasesBroken=NO","speedWanted=NoHypos","comment=Including Related Terms","elimEnable=NO","constraintMinTerms=2"
15	..nlpx "query=((prompt or prompts or cue or cues)):ti,ab,kw","desiredResults=10000","minHitsDivisor=7","permitHyponyms=NO","lowestVocabularySearchLevel=none","phrasesBroken=NO","speedWanted=NoHypos","comment=Including Related Terms","elimEnable=NO","constraintMinTerms=2"
16	..nlpx "query=("hand signal" or "hand signals" or "verbal signal" or "verbal signals"):ti,ab,kw","desiredResults=10000","minHitsDivisor=7","permitHyponyms=NO","lowestVocabularySearchLevel=none","phrasesBroken=NO","speedWanted=NoHypos","comment=Including Related Terms","elimEnable=NO","constraintMinTerms=2"
17	..nlpx "query=((sbar or callout* or call out* or checkback* or check back* or DESC)):ti,ab,kw","desiredResults=10000","minHitsDivisor=7","permitHyponyms=NO","lowestVocabularySearchLevel=none","phrasesBroken=NO","speedWanted=NoHypos","comment=Including Related Terms","elimEnable=NO","constraintMinTerms=2"
18	..nlpx "query=((communication NEAR/3 (closed or loop*))):ti,ab,kw","desiredResults=10000","minHitsDivisor=7","permitHyponyms=NO","lowestVocabularySearchLevel=none","phrasesBroken=NO","speedWanted=NoHypos","comment=Including Related Terms","elimEnable=NO","constraintMinTerms=2"
19	..nlpx "query=("closing the loop"):ti,ab,kw","desiredResults=10000","minHitsDivisor=7","permitHyponyms=NO","lowestVocabularySearchLevel=none","phrasesBroken=NO","speedWanted=NoHypos","comment=Including Related Terms","elimEnable=NO","constraintMinTerms=2"
20	..nlpx "query=("pass the baton"):ti,ab,kw","desiredResults=10000","minHitsDivisor=7","permitHyponyms=NO","lowestVocabularySearchLevel=none","phrasesBroken=NO","speedWanted=NoHypos","comment=Including Related Terms","elimEnable=NO","constraintMinTerms=2"
21	..nlpx "query=("crew resource"):ti,ab,kw","desiredResults=10000","minHitsDivisor=7","permitHyponyms=NO","lowestVocabularySearchLevel=none","phrasesBroken=NO","speedWanted=NoHypos","comment=Including Related Terms","elimEnable=NO","constraintMinTerms=2"
22	..nlpx "query=("two challenge rule"):ti,ab,kw","desiredResults=10000","minHitsDivisor=7","permitHyponyms=NO","lowestVocabularySearchLevel=none","phrasesBroken=NO","speedWanted=NoHypos","comment=Including Related Terms","elimEnable=NO","constraintMinTerms=2"
23	..nlpx "query=("speak up" or "speaking up"):ti,ab,kw","desiredResults=10000","minHitsDivisor=7","permitHyponyms=NO","lowestVocabularySearchLevel=none","phrasesBroken=NO","speedWanted=NoHypos","comment=Including Related Terms","elimEnable=NO","constraintMinTerms=2"
24	8 or 9 or 10 or 11 or 12 or 13 or 14 or 15 or 16 or 17 or 18 or 19 or 20 or 21 or 22 or 23
25	6 and 24
26	7 or 25
27	..nlpx "query=((aviation or aerospace or aeronautic* or cockpit or military or aviator* or flight personnel or pilots or fighter pilot* or battlefield* or power plant*)):ti,ab,kw","desiredResults=10000","minHitsDivisor=7","permitHyponyms=NO","lowestVocabularySearchLevel=none","phrasesBroken=NO","speedWanted=NoHypos","comment=Including Related Terms","elimEnable=NO","constraintMinTerms=2"
28	..nlpx "query=MeSH descriptor: [Aviation] explode all trees","desiredResults=10000","minHitsDivisor=7","permitHyponyms=NO","lowestVocabularySearchLevel=none","phrasesBroken=NO","speedWanted=NoHypos","comment=Including Related Terms","elimEnable=NO","constraintMinTerms=2"
29	..nlpx "query=MeSH descriptor: [Military Personnel] explode all trees","desiredResults=10000","minHitsDivisor=7","permitHyponyms=NO","lowestVocabularySearchLevel=none","phrasesBroken=NO","speedWanted=NoHypos","comment=Including Related Terms","elimEnable=NO","constraintMinTerms=2"
30	..nlpx "query=MeSH descriptor: [Nuclear Power Plants] explode all trees","desiredResults=10000","minHitsDivisor=7","permitHyponyms=NO","lowestVocabularySearchLevel=none","phrasesBroken=NO","speedWanted=NoHypos","comment=Including Related Terms","elimEnable=NO","constraintMinTerms=2"
31	..nlpx "query=MeSH descriptor: [Operating Rooms] explode all trees","desiredResults=10000","minHitsDivisor=7","permitHyponyms=NO","lowestVocabularySearchLevel=none","phrasesBroken=NO","speedWanted=NoHypos","comment=Including Related Terms","elimEnable=NO","constraintMinTerms=2"
32	..nlpx "query=((operating NEAR/2 (room* or theatre*))):ti,ab,kw","desiredResults=10000","minHitsDivisor=7","permitHyponyms=NO","lowestVocabularySearchLevel=none","phrasesBroken=NO","speedWanted=NoHypos","comment=Including Related Terms","elimEnable=NO","constraintMinTerms=2"
33	..nlpx "query=(high risk industr*):ti,ab,kw","desiredResults=10000","minHitsDivisor=7","permitHyponyms=NO","lowestVocabularySearchLevel=none","phrasesBroken=NO","speedWanted=NoHypos","comment=Including Related Terms","elimEnable=NO","constraintMinTerms=2"
34	..nlpx "query=MeSH descriptor: [Intraoperative Period] explode all trees","desiredResults=10000","minHitsDivisor=7","permitHyponyms=NO","lowestVocabularySearchLevel=none","phrasesBroken=NO","speedWanted=NoHypos","comment=Including Related Terms","elimEnable=NO","constraintMinTerms=2"
35	..nlpx "query=(((perioperative or intraoperative) NEAR/2 (period or setting or environment))):ti,ab,kw","desiredResults=10000","minHitsDivisor=7","permitHyponyms=NO","lowestVocabularySearchLevel=none","phrasesBroken=NO","speedWanted=NoHypos","comment=Including Related Terms","elimEnable=NO","constraintMinTerms=2"
36	..nlpx "query=((during or undergoing) NEAR/3 surgery):ti,ab,kw","desiredResults=10000","minHitsDivisor=7","permitHyponyms=NO","lowestVocabularySearchLevel=none","phrasesBroken=NO","speedWanted=NoHypos","comment=Including Related Terms","elimEnable=NO","constraintMinTerms=2"
37	..nlpx "query=MeSH descriptor: [Surgical Procedures, Operative] explode all trees","desiredResults=10000","minHitsDivisor=7","permitHyponyms=NO","lowestVocabularySearchLevel=none","phrasesBroken=NO","speedWanted=NoHypos","comment=Including Related Terms","elimEnable=NO","constraintMinTerms=2"
38	..nlpx "query="surgical team*":ti,ab","desiredResults=10000","minHitsDivisor=7","permitHyponyms=NO","lowestVocabularySearchLevel=none","phrasesBroken=NO","speedWanted=NoHypos","comment=Including Related Terms","elimEnable=NO","constraintMinTerms=2"
39	27 or 28 or 29 or 30 or 31 or 32 or 33 or 34 or 35 or 36 or 37 or 38
40	26 and 39
41	limit 40 to yr="2019 - 2022"
Scopus	
1	( ( ( ( TITLE ( team* ) ) OR ( TITLE-ABS ( team* W/2 ( behaviour OR behavior OR situation OR performance ) ) ) OR ( TITLE-ABS-KEY ( "teamwork" OR "team member*" ) ) ) ) AND ( ( TITLE ( communication ) ) OR ( TITLE-ABS-KEY ( communication AND strateg* ) OR ( TITLE-ABS ( ( nonverbal OR nonverbal ) W/3 ( communicat* OR interaction* ) ) ) OR ( KEY ( "Verbal Behavior" ) ) OR ( TITLE-ABS-KEY ( team* W/3 training ) ) OR ( TITLE-ABS-KEY ( checklist* OR "check list*" ) ) OR ( TITLE-ABS-KEY ( script* OR whiteboard* OR toolkit* ) ) OR ( TITLE-ABS-KEY ( prompt OR prompts OR cue OR cues OR "visual signal*" OR "hand signal" ) ) OR ( TITLE-ABS-KEY ( sbar OR callout* OR "call out*" OR checkback* OR "check back*" OR desc ) ) OR ( title ABS ( situation AND background AND assessment AND recommendation ) ) OR ( TITLE-ABS ( communication W/3 ( closed OR loop* ) ) ) OR ( TITLE-ABS ( "clos* the loop" ) ) OR ( TITLE-ABS ( "pass the baton" ) ) OR ( TITLE-ABS ( "two challenge rule" ) ) OR ( TITLE-ABS ( "speak up" OR "speaking up" ) ) OR ( TITLE-ABS-KEY ( "crew resource" ) ) ) ) OR ( TITLE-ABS-KEY ( "team* communication" ) ) ) AND ( ( TITLE-ABS-KEY ( aviation OR aerospace OR aeronautic* OR cockpit OR military OR aviator* OR pilots OR "fighter pilot" OR "flight personnel" OR battlefield* OR "power plant*" ) ) OR ( TITLE-ABS-KEY ( operating W/2 ( room* OR theatre* ) ) . ) OR ( TITLE-ABS ( "surgical team*" ) ) OR ( TITLE-ABS ( ( during OR undergoing ) W/3 surgery ) ) OR ( TITLE-ABS ( ( perioperative OR intraoperative ) W/2 ( period OR setting OR environment ) ) ) OR ( TITLE-ABS ( "high risk" W/2 ( industr* OR environment* OR setting* ) ) ) ) AND ( LIMIT-TO ( PUBYEAR , 2022 ) OR LIMIT-TO ( PUBYEAR , 2021 ) OR LIMIT-TO ( PUBYEAR , 2020 ) OR LIMIT-TO ( PUBYEAR , 2019 ) )

**Table 4:** List and characteristics of included studies (N=249)

First author, year	Country	Study design	Sample	Setting	Type of intervention	Name of intervention
Askarian et al., 2011	Iran	Before and after interventional study	144 surgical cases	Operating room	Bundle/Checklists	Surgical Safety Checklist
Bartz-Kurycki et al., 2017	United States	Observational cohort study	603 surgical cases	Operating room	Bundle/Checklists	Debriefing
Bereknyei Merrell et al., 2018	United States	Case report	6 healthcare professionals (anesthesia attending, surgical resident, surgical attending, surgical technician, circulating nurse, nurse anesthetist)	Operating room	Bundle/Checklists	Emergency manuals
Calland et al., 2011	United States	Randomized controlled trial	47 surgical cases, 10 surgical attendings	Operating room	Bundle/Checklists	Surgical Safety Checklist
Chen et al., 2013	United States	Literature review	N/A	Operating room	Bundle/Checklists	Cerebral aneurysm checklist
Cumin et al., 2017	New Zealand	Observational cohort study	120 healthcare professionals (20 teams of 6: consultant surgeon, surgical registrar, anaesthetist, anaesthetic technician, circulating nurse, scrub nurse)	Operating room	Bundle/Checklists	Information probes (briefing notes)
Dabholkar et al., 2018	India	Prospective, non-randomised, comparative study	37 healthcare professionals (15 surgeons, 14 anaesthetists, 8 nurses)	Operating room	Bundle/Checklists	Surgical Safety Checklist
De Muinck Keizer et al., 2017	Netherlands	Experimental study	1255 surgical procedures, 33 healthcare professionals (17 surgical residents/attendings,16 radiographers)	Operating room	Protocol	Uniform C-arm communication terminology
de Vries et al., 2011	Netherlands	Retrospective claim record review	294 surgical malpractice claims	Operating room	Bundle/Checklists	SURgical PAtient Safety System (SURPASS) checklist
Dixon et al., 2016	United States	Observational cohort study	Baseline: 39 healthcare professionals (8 anesthesia providers, 9 circulating nurses, 11 scrub technicians, 11 surgeons) Post-intervention: 42 healthcare professionals (10 anesthesia providers, 14 circulating nurses, 7 scrub technicians, 11 surgeons)	Operating room	Bundle/Checklists	Multimedia (video)-based checklist for time-out
Dobbie et al., 2019	United States	Observational cohort study	680 preoperative audits	Operating room	Audit & Feedback	Remote Audiovisual Observation
El-Shafy et al., 2018	United States	Observational cohort study	89 trauma activation videos involving surgical attending or fellow, surgical resident, emergency medicine attending, fellow, or resident	Trauma room	Protocol	Closed Loop Communication
Erestam et al., 2017	Sweden	Before and after interventional study	150 healthcare professionals (surgeons, anesthesiologists, scrub nurses, nurse anaesthetists, nurse assistants)	Operating room	Bundle/Checklists	Surgical Safety Checklist
Everett et al., 2017	Canada	Randomized controlled trial	56 simulation encounters involving OR teams comprised of a surgeon, anaesthetist, and three nurses	Operating room	Bundle/Checklists	Critical event checklists
Fang et al., 2018	United States	Observational cohort study	100 healthcare professionals (56 interns, 30 residents, 14 attendings)	Internal medicine	Other	Hands Free Communication Devices (HFCD)
Fernandes et al., 2015	Canada	Case series	4 surgical cases	Operating room	Protocol	Transcatheter aortic valve implantation (TAVI) protocol
Freundlich et al., 2015	United States	Observational cohort study	166 time-outs involving anesthesia team, surgeons, nurses, scrub technicians	Operating room	Audit & Feedback	Time-out
Gillespie et al., 2010	Australia	Qualitative interview study	16 healthcare professionals (4 physicians, 3 nurse managers, 9 nurses)	Operating room	Bundle/Checklists	Time-out
Goff et al., 2018	United States	Observational cohort study	115 healthcare professionals (50 surgical attendings, 65 surgical residents)	Operating room	Protocol	Navigational Grid
Henrickson et al., 2009	United States	Observational cohort study	56 healthcare professionals (surgical assistants, surgical technicians, circulating nurses, perfusionists, nurse anesthetists)	Operating room	Protocol	Preoperative Briefing Protocol for Cardiovascular Surgery
Hicks et al., 2014	United States	Review	N/A	Operating room	Bundle/Checklists	Operating room briefings
Hunter et al., 2017	United States	Observational cohort study	23 surgical cases involving circulating nurses, surgical technicians, surgical assistants, anesthesia team members	Operating room	Protocol	SBAR tool
Julia et al., 2017	France	Interventional cohort study	204 anesthesia handovers involving residents and nurse anesthetists	Operating room	Bundle/Checklists	Intraoperative handover training and checklist
Kearns et al., 2011	United Kingdom	Observational cohort study	53 healthcare professionals (17 midwives, 8 auxiliaries, 8 obstetric trainees, 8 anaesthetic trainees, 5 anaesthetic nurses, 4 anaesthetic consultants, 3 consultant obstetricians)	Operating room	Bundle/Checklists	Surgical Safety Checklist
Kozusko et al., 2016	United States	Observational cohort study	4,453 surgical cases involving a surgeon, anesthesia care provider, circulating nurse, preoperative nurse, and relief nurse	Operating room	Bundle/Checklists	Surgical time-out patient-focused model
Lingard et al., 2008	Canada	Observational cohort study	128 health care professionals (11 general surgeons, 24 surgical trainees, 41 operating room nurses, 28 anesthesiologists, 24 anesthesia trainees)	Operating room	Bundle/Checklists	Checklist and Briefing
Lingard et al., 2011	Canada	Retrospective preintervention/postintervention study	340 surgical cases, 243 healthcare professionals (11 surgeons, 48 surgical residents and fellows, 87 operating room nurses, 3 nursing trainees, 60 staff anesthesiologists, 26 anesthesia residents and fellows, 3 respiratory therapists, 5 technical assistants)	Operating room	Bundle/Checklists	Preoperative team checklist
Lingard et al., 2005	Canada	Observational cohort study	22 surgical cases, 33 healthcare professionals (8 surgeons, 8 staff anesthesiologists, 4 anesthesia residents, 3 surgical residents, 10 nurses)	Operating room	Bundle/Checklists	Preoperative team briefing
Low et al., 2013	United States	Cross-sectional study	29 healthcare professionals	Operating room	Bundle/Checklists	Flow checklist
MacDougall-Davis et al., 2016	United Kingdom	Observational cohort study	32 healthcare professionals (8 teams of 4 anaesthetic trainees and "go-betweens")	Operating room	Protocol	Traffic Lights tool
Mainthia et al., 2012	United States	Observational cohort study	240 surgical cases involving surgical and anesthesia residents, fellows, and attendings; registered nurse anesthetists, scrub nurses, circulating nurses; OR technicians	Operating room	Bundle/Checklists	Electronic Whiteboard Checklist
Makary et al., 2006	United States	Review	N/A	Operating room	Bundle/Checklists	OR Briefing
Marshall et al., 2016	Australia	Randomized controlled trial	72 healthcare professionals (24 teams of 3, consisting of a consultant anesthetist, an anesthetic trainee and anesthetic assistant)	Operating room	Clinical Practice Guideline	Guidelines for the management of peri-operative severe allergic reactions
Masiglat et al., 2016	United States	Short Report	N/A	Operating room	Bundle/Checklists	Wilmer Hand-off Communication Tool
McFerran et al., 2005	United States	Short Report	N/A	Perinatal care	Bundle/Checklists	Perinatal Patient Safety Project
Norton et al., 2010	United States	Short Report	N/A	Operating room	Bundle/Checklists	Pediatric Surgical Safety Checklist
O'Connor et al., 2013	Ireland	Cross-sectional study	107 healthcare professionals (41 surgeons, 33 anaesthetists, 33 nurses)	Operating room	Bundle/Checklists	Surgical Safety Checklist
Overdyk et al., 2016	United States	Cluster randomized controlled trial	2,693 surgical cases involving surgeons, anesthesia providers, nurses, support staff	Operating room	Audit & Feedback	Remote video auditing (RVA)
Papaconstantinou et al., 2013	United States	Cross-sectional study	437 healthcare professionals (153 nurses, 104 anesthesia providers, 180 surgeons)	Operating room	Protocol	Surgical Safety Checklist
Papaspyros et al., 2010	United Kingdom	Retrospective case review	118 surgical cases, 15 healthcare professionals (anaesthetists, perfusionists, scrub nurses, technicians)	Operating room	Bundle/Checklists	Briefing and debriefing checklist
Pian-Smith et al., 2009	United States	Observational cohort study	40 anesthesia trainees	Operating room	Bundle/Checklists	Two-challenge rule
Pickering et al., 2013	United Kingdom	Observational cohort study	26 surgical cases	Operating room	Bundle/Checklists	Surgical Safety Checklist
Pulido et al., 2017	United States	Randomized controlled trial	17 surgeons	Operating room	Protocol	Surgeon's verbal intervention
Ragusa et al., 2016	United States	Review	N/A	Operating room	Bundle/Checklists	Surgical Safety Checklist
Randmaa et al., 2014	Sweden	Randomized controlled trial	169 healthcare professionals (practical nurses, registered nurses, physicians)	Operating room, intensive care unit, post-anesthesia care unit	Protocol	SBAR tool
Rhee et al., 2017	United States	Observational cohort study	1,610 surgical time- outs and debriefs	Operating room	Bundle/Checklists	TeamSTEPPS
Santana et al., 2016	Brazil	Cross-sectional study	472 health professionals (surgeons, anesthesiologists, surgical technologists, nurses, nursing technicians and nursing assistants, resident physicians, medical and nursing students, heads of medical and nursing services)	Operating room	Bundle/Checklists	Surgical Safety Checklist
Schwendimann et al., 2019	Switzerland	Observational cohort study	104 on-site observations, 11 healthcare professionals (6 surgeons and anaesthesiologists, 5 operating room nurses and nurse anaesthetists)	Operating room	Bundle/Checklists	Surgical Safety Checklist
Webster et al., 2006	United States	Randomized controlled trial	36 healthcare professionals	Operating room	Protocol	Scripted/Automatic Speech Communication
Weingessel et al., 2017	Austria	Observational cohort study	18,081 surgical procedures	Operating room	Bundle/Checklists	Time-out
West et al., 2012	United States	Observational cohort study	47 healthcare professionals (26 registered nurses, 12 licensed vocational nurses, 9 nurse anesthetists)	Cardiac medicine unit	Protocol	Sterile Cockpit Rule
Wright et al., 2016	United States	Observational cohort study	30 surgical cases	Operating room	Education	Educational noise reduction intervention (No interruption zones)
Zeeni et al., 2014	United States	Observational cohort study	548 surgical patients	Operating room	Protocol	High Risk Spine Protocol
Faiz et al., 2019	Pakistan	Before and after interventional study	60 patient transfers	Intensive Care Unit	Bundle/Checklists	Standardized patient handover process
Carpini et al., 2020	Australia	Cross-sectional study	46 registered nurses from short-stay surgical units	Pre-operative	Protocol	Multidisciplinary team briefings (MDTB)
Tankimovich et al., 2020	United States	Pilot study	20 participants (trainees)	Outpatient setting	Education	Interprofessional education (IPE) and teamwork (TW) simulation exercise using TeamSTEPPS Pocket Guide
Roig et al., 2020	Argentina	Before and after interventional study	158 pre-intervention and 124 post-intervention handoff assessments	Pediatric unit	Education	I-PASS
Wunder et al., 2020	United States	Quantitative, descriptive study	34 student registered nurse anesthetists	Operating room	Education	Operating Room Fire Simulation using Magic Leap OneTM augmented reality headsets
Staines et al., 2020	Switzerland	Pre-and-post observational study	90 completed questionnaires	Maternity ward	Education	TeamSTEPPS teamwork improvement concept
Loesche et al., 2020	United States	Pre-and-post observational study	19 participants	Instrument-processing department	Protocol	Daily huddles
DeBrún et al., 2020	Ireland	Cross-sectional study	Four heterogeneous healthcare teams	Heterogeneous healthcare teams, ranging in size from small cross-organisational teams to large unit-based teams in large urban teaching hospitals	Education	The Collective Leadership for Safety Cultures (Co-Lead) programme
Valdes et al., 2021	United States	Before and after interventional study	10 nursing students	Escape Room	Education	Escape Room Simulation
Tervajärvi et al., 2021	Finland	Prospective, non-randomised, comparative study	21 participants (trainees)	Emergency Department	Education	Student-LED interprofessional sequential simulation
Raîche et al., 2021	Canada	Prospective observational study	22 simulation cases	Operating room	Education	In situ simulation sessions
Rojo‐rojo et al., 2021	Spain	Mixed pilot study (qualitative/quantitative) with three phases and a pre-post intragroup quasi-experimental study	12 simulation participants	Intensive Care Unit; Emergency Department	Education	High Fidelity Simulation
Lee et al., 2021	United States	Prospective pre-post cohort study	104 surgical staff members	Operating room	Education	Four reinforcement activities
Ulmer et al., 2022	Switzerland	Pre-and-post observational study	15 nurses	Intensive Care Unit	Education	In situ simulation team training focused on communication
Undre et al., 2007	UK	Cross-sectional study	50 urology procedures	Operating room	Audit & Feedback	Observational Teamwork Assessment for Surgery (OTAS)
Bethune et al., 2011	UK	Before and after interventional study	100 questionnaires completed by all OR team members	Operating room	Protocol	Briefings and debriefings
Whyte et al., 2008	Canada	Prospective, non-randomised, comparative study	302 preoperative team briefings	Operating room	Bundle/Checklists	Preoperative team briefing
Marzano et al., 2016	United States	Pre-and-post observational study	12 simulation sessions	Operating room	Protocol	Birth Center Pager (BCP)
Størkson et al., 2016	Norway	Cross-sectional study	268 (54% of total 501 completed forms)	Operating room; Trauma room; Internal medicine; Perinatal care; Intensive Care Unit; Post-anesthesia care unit; Cardiac medicine unit	Audit & Feedback	Care Process Self-Evaluation Tool (CPSET)
Kvarnström et al., 2018	Sweden	Qualitative ethnographic study	89 health professionals	Surgical ward	Protocol	Introduction of NPs into surgical ward teams
Funk et al., 2016	United States	Pre-and-post observational study	Samples of 52 pre-implementation and 51 post-implementation handover interaction	Post-anesthesia care unit	Protocol	Introductions, Situation,Background, Assessment, Recommendations, and Questions (ISBARQ) checklist
Collazos et al., 2013	Colombia	Cross-sectional study	A total of 246 patients were surveyed during February andMarch 2011, 29% females and 71% males. The mean age was48.5 years; the age range was between 18 and 88 years	Operating room; Post-anesthesia care unit	Bundle/Checklists	WHO's surgical checklist
Reed et al., 2016	UK	Prospective, non-randomised, comparative study	92 procedures	Operating room	Bundle/Checklists	Audio delivery of the Surgical Safety Checklist (SSC)
Vyas et al., 2013	United States	Before and after interventional study	N/A	Operating room	Protocol	Global Smile Foundation Emergency Response Protocol
Yamada et al., 2015	United States	Prospective, randomised, comparative study	13 simulation scenarios	Perinatal care	Education	Standardized Communication Techniques
Yule et al., 2015	United States	Randomised controlled trial	16 surgical residents	Operating room	Education	Non-Technical Skills for Surgeons (NOTSS) behavior observation system coaching
Skelton et al., 2016	Rwanda	Before and after interventional study	20 participants	Operating room	Education	Anesthetists’ Non-technical Skills (ANTS) training using low-cost high psychological fidelity simulation with debriefing
Flin et al., 2004	UK	Prospective, non-randomised, comparative study	8 simulated cases (number of participants not specified)	Operating room	Education	Crisis Avoidance Resource Management for Anaesthetists (CARM-A)
Sudikoff et al., 2009	United States	Randomised crossover trial	16 residents	Operating room	Education	High-fidelity medical simulation
Weaver et al., 2010	United States	Before and after interventional study	N/A	Operating room	Education	TeamSTEPPS Training Program
Ostermann et al., 2010	Germany	Before and after interventional study	121 participants (77 staff members and 44 patients’ relatives)	Integrative hospital for neurological rehabilitation	Education	Team-building process consisted of didactic instruction and training in problem-solving, teambuilding and constructive conflict resolution.
Capella et al., 2010	United States	Before and after interventional study	A convenience sample (n=33) trauma resuscitations before training, and (n=40) post training	Trauma room	Education	TeamSTEPPS training, augmented by simulation
Sculli et al., 2012	United States	Training design	N/A	Operating room; Internal medicine	Education	Clinical Crew Resource Management (CCRM)
Johnson et al., 2012	United States	Before and after interventional study	809 participants	Operating room	Education	Perioperative Teamwork Education Program (Safety training program focusing on Crew Resource Management, TeamSTEPPS, and communication techniques)
Willaert et al., 2012	Belgium	Prospective, observational study	18 cases	Operating room	Education	Patient-specific virtual reality rehearsal
Wheeler et al., 2013	United States	Non-randomised experimental study	112 simulations	Internal medicine	Education	Simulations
Kilday et al., 2013	United States	Pre-and-post observational study	29 neonatal rapid response team members participated	NICU	Education	Combined team training program (combining evidence-based education, team concepts and simulation training)
Abdelshehid et al., 2013	United States	Pre-and-post observational study	Nine urology residents, 7 anesthesia residents, and 2 CRNA participated in the 9-study scenario presentation	Operating room	Education	Simulation-based team training (SBTT)
Farra et al., 2014	United States	Prospective, non-randomised, comparative study	18 nursing students	A midsize public university	Education	Disaster Triage Virtual Reality Simulation
Perkins et al., 2015	United States	Prospective, non-randomized comparative study	22 surgical technicians and operating room nurses	Operating room	Education	American College of Surgeons’ Advanced Trauma Operative Management (ATOM) course
Arora et al., 2015	UK	Before and after interventional study	185 residents from 5 hospitals	Surgical wards	Education	Simulation-based training
Lisbon et al., 2016	United States	Pre-and-post observational study	113 members of an academic emergency department	Emergency Department	Education	TeamSTEPPS educational strategy
Hoang et al., 2016	United States	Before and after interventional study	55 participants (11 teams)	Operating room	Education	Shipboard Surgical Trauma Training Course (S2T2C)
James et al., 2016	United States	Observational case study and questionnaire of participants in a cross-sectional analysis	23 learners	Haematology-oncology unit	Education	Simulation-based team training scenarios
Xu et al., 2016	France	Prospective, non-randomised, comparative study	28 participants	Operating room	Education	Xperience™ Team Trainer (XTT)
Chalwin et al., 2016	Australia	Cross-sectional study	96 participants	Conference (ANZICS: The Deteriorating Patient Conference)	Education	ANZICS RRT Training Program
Savage et al., 2017	Sweden	Case study	153 managers and staff	Operating room	Protocol	Crew Resource Management (CRM) safety program
Clapper et al., 2018	United States	Quantitative pre-test and post-test study	109 participants (16 groups)	Internal medicine unit	Education	Code team course
Chamberland et al., 2018	Canada	Randomised controlled trial	60 health‐care professionals	Intensive Care Unit	Audit & Feedback	simulation‐based learning - debriefing content
Bian et al., 2019	Netherlands	Concept study	N/A	Operating room	Environment improvement	Automatic Integration of Medical Information (AIMI)
Fukushima et al., 2018	Japan	Randomized, controlled, prospective pilot study	33 medical students	Simulation Center	Education	Team Strategies and Tools to Enhance Performance and Patient Safety (TeamSTEPPS) program
Adams et al., 2004	United States	Prospective, non-randomised, comparative study	96 surgical cases	Operating room	Education	Six Sigma
McKoin et al., 2010	United States	Pre-and-post observational study	530 surgical staff members	Operating room	Education	Crew Resource Management (CRM) Team Training
Catchpole et al., 2010	UK	Prospective, non-randomised, comparative study	112 operations (51 before and 61 after intervention)	Operating room	Education	Aviation-style team training (+Theatre Aide Memoir)
Spence et al., 2011	Canada	Cross-sectional study	130 students visiting 65 Operating rooms	Operating room	Audit & Feedback	Student-observed surgical safety practices + WHO surgical checklist
Mayer et al., 2011	United States	Pre-and-post observational study	12 attendings, 157 nursing staff, and 90 respiratory therapists participated.	Intensive Care Unit; Post-anesthesia care unit	Education	TeamSTEPPS
Sculli et al., 2011	United States	Short Report	54025 employees (147 locations)	Veterans Health Administration	Audit & Feedback	National Center for Patient Safety (NCPS) patient safety culture survey
Paull et al., 2013	United States	Pre-and-post observational study	334 perioperative surgical staff	Operating room; Intensive Care Unit	Education	point-of-care simulation-based team training curriculum
Harvey et al., 2013	United States	Prospective, non-randomised, comparative study	N/A	Trauma room	Education	TeamSTEPPS simulation-based training
Fernandez et al., 2013	United States	Randomized comparison trial	231 participants (medical students and residents)	Emergency Room	Education	Computer-based teamwork process training intervention
Howe et al., 2014	United States	Before and after interventional study	15 staff members	Long-term care facility	Bundle/Checklists	TeamSTEPPS Long-Term Care (LTC) Team Talk
Braham et al., 2014	UK	Before and after interventional study	54 cases total. 20 cases (pre, old checklist) vs 34 cases (post, modified checklist)	Operating room	Bundle/Checklists	A modified World Health Organization (WHO) Safety Checklist
Tibbs et al., 2014	United States	Pre-and-post observational study	18 surgical team members	Operating room	Bundle/Checklists	Team Strategies & Tools to Enhance Performance and Patient Safety (TeamSTEPPS)
Korkiakangas et al., 2015	UK	Non-randomised experimental study	Two scenarios	Operating room	Education	Video-Supported Simulation for Interactions in the Operating Theatre (ViSIOT)
Copeland et al., 2016	United States	Before and after interventional study	Forty-six (27 females, 19 males) of the 155 staff members (30%) responded to the pre-implementation survey: 32 RNs, 10 MDs/DOs, two psychiatric assessors, one PA, and one critical care technician (CCT). Thirty-seven (24 females, 13 males) of the 192 staff members (19%) responded to the mid-implementation survey: 26 RNs, seven MDs/DOs, two CCTs, and two unit secretaries. Thirty-three (21 females, 12 males) of the 173 staff members (19%) responded to the post-implementation survey: 25 RNs, four MD/DOs, three unit secretaries, and one CCT.	Trauma room (Emergency department)	Protocol	Post-Code Pause
Yang et al., 2016	China	Before and after interventional study	168 Handovers [Nurses (n=77), resident physicians (n=20), intensive care specialists (n=10) and respiratory therapists (n=2) of the ICU as well as all neurosurgeons(n=34) and anaesthetists (n=13)]	Intensive Care Unit	Protocol	Handover protocol
Parush et al., 2017	Canada	Pre-and-post observational study	13	Trauma room	Technological Cognitive Aid	Situation Display
Finch et al., 2019	United States	Before and after interventional study	32 OR rooms (exact number not collected)	Operating room	Education	Coaches for improving SSC debriefing
Small et al., 1999	United States	Non-randomised experimental study	15 patient scenario concepts, 15 participants	Emergency Room	Education	High-fidelity Simulation Team Training Course for Emergency Medicine
Morey et al., 2002	United States	Pre-and-post observational study	1058 physicians, nurses, and technicians (374 control group, 684 experimental group)	Emergency Department	Education	EmergencyTeam Coordination Course (ETCC)
Blum et al., 2005	United States	Cross-sectional study	22 pilot teams	Operating room	Audit & Feedback	Anesthesia Crisis Resource Management (ACRM)
Leming-Lee et al., 2005	United States	Prospective, non-randomised, comparative study	737 providers were trained and provided feedback	Operating room	Education	Crew Resource Management (CRM)
Awad et al., 2005	United States	Pre-and-post observational study	N/A	Operating room	Education	Medical Team Training (MTT)
Undre et al., 2007	UK	Prospective, non-randomised, comparative study	80 participants (20 teams)	Operating room	Education	Multidisciplinary Crisis Simulations
Catchpole et al., 2007	UK	Pre-and-post observational study	48 surgical cases	Operating room	Audit & Feedback	Direct observation
Flin et al., 2007	UK	Cross-sectional study	18 surgeons returned the evaluation form	Operating room	Education	Non-Technical Skills for Surgeons (NOTSS) training course
Sax et al., 2009	United States	Prospective, non-randomised, comparative study	857 participants	Operating room	Education	Crew resource management training intervention
Willaert et al., 2010	UK	Before and after interventional study	One surgical case (one patient)	Simulation centre	Education	Patient-specific virtual reality simulation
Neily et al., 2010	United States	Descriptive analysis	32 facilities	Operating room	Education	Learning session
Gore et al., 2010	United States	Before and after interventional study	600 surveys	Operating room	Education	Crew Resource Management (CRM) training
Wolf et al., 2010	United States	Prospective, non-randomised, comparative study	4,863 cases	Operating room	Education	Medical Team Training (MTT)
Polack et al., 2010	United States	Prospective, non-randomised, comparative study	149 trauma personnel	Trauma room	Education	Communication curriculum
Joy et al., 2011	United States	Prospective, interventional study	79 patient handovers	Operating room; Intensive Care Unit	Protocol	Standardized handover protocol
Steinemann et al., 2011	United States	Before and after interventional study	137 multidisciplinary trauma team members	Trauma room	Education	In situ, multidisciplinary, simulation-based teamwork training
Deering et al., 2011	United States	Before and after interventional study	153 patient safety reports submitted (94 before implementation, 59 after implementation)	Operating room; Trauma room	Education	TeamSTEPPS
Suva et al., 2012	Switzerland	Prospective, non-randomised, comparative study	99 participants	Operating room	Education	Crew Resource Management (CRM) program
Hoang et al., 2013	United States	Prospective, non-randomised, comparative study	N/A	Trauma room	Education	Deployment Fleet Surgical Team Training
Böhmer et al., 2013	Germany	Pre-and-post observational study	A survey of 99 co-workers	Operating room; Trauma room	Bundle/Checklists	"Safety checklist", an adaptation of 'safe surgery checklist’ of the WHO
Passauer-Baierl et al., 2014	Germany	Prospective, non-randomised, comparative study	11 surgical cases	Operating room	Audit & Feedback	Observational Teamwork Assessment for Surgery Tool (OTAS-D)
Kellicut et al., 2014	US study (but took place in Iraq)	Before and after interventional study	220 personnel completed the training, of which 61 completed the survey.	Operating room; Trauma room	Education	Surgical Team Assessment Training (STAT)
Fouilloux et al., 2014	France	Prospective, non-randomised, comparative study	4 participants (1 team)	Operating room	Education	Team training in management of adverse acute events occurring during cardiopulmonary bypass procedure
Hughes et al., 2014	United States	Before and after interventional study	160 personnel (132 surveys and 38 observations post)	Trauma room	Protocol	Crew Ressource Management (CRM)
Morgan et al., 2015	UK	Controlled interrupted time series study	2,221 patients and 94 surgical cases (1,121 patients before intervention and 1,100 after intervention; 44 operations observed before and 50 after)	Operating room	Education	Teamwork training (SOP and CRM-style team training)
Phitayakorn et al., 2015	United States	Prospective, non-randomised, comparative study	5 intraoperative teams	Operating room	Simulation	Anesthesiologists’ Non-Technical Skills (ANTS), Scrub Practitioners List of Intra-operative Non-Technical Skills (SPLINTS), Non-Technical Skills for Surgeons (NOTSS), Objective Teamwork Assessment System (OTAS), and an evidence-based MH checklist.
Nakarada-Kordic et al., 2016	New Zealand	Prospective, non-randomised, comparative study	20 complete OR teams (comprising 120 healthcare professionals in total)	Operating room	Cognitive aid	Computer-based card sorting tool (Momento)
Norton et al., 2016	United States	Before and after interventional study	196 multidisciplinary operating room clinicians responded to the survey	Operating room	Bundle/Checklists	Pediatric Surgical Safety Checklist
Stephens et al., 2016	UK	Prospective, non-randomised, comparative study	130 staff	Operating room	Education	Interprofessional training course in crises and human factors
Weller et al., 2016	New Zealand	Before and after interventional study	437 general surgical cases (224 cases before and 213 cases after MORSim)	Operating room	Education	Multidisciplinary Operating Room Simulation (MORSim)
Rao et al., 2016	United States	Prospective cohort study with pretesting or posttesting	15 postgraduate year 1 general surgery residents	Operating room	Education	Team-based tasks designed to teach communication and teamwork
Henderson et al., 2016	United States	Pre-and-post observational study	68 nurses	Operating room; Intensive Care Unit; Post-anesthesia care unit	Protocol	Pop-form (postoperative communication system)
Earle et al., 2017	United States	Pre-and-post observational study	N/A	Operating room	Protocol	Circulate, Scrub and Technical AssistanceTeam (C-STAT)
Stewart-Parker et al., 2017	UK	Before and after interventional study	68 healthcare professionals	Operating room	Education	S-TEAMS Course
Khademian et al., 2018	Iran	Quasi-Experimental	60 students (45 anesthesia and 15 operating room nursing students)	Operating room	Education	Teamwork Training Workshop
Caskey et al., 2017	United States	Pre-and-post observational study	9 surgical cases (9 PGY-1 residents)	Operating room	Education	Laparoscopic team-based task training for nontechnical skills
Rao et al., 2017	United States	Prospective, non-randomised, comparative study	53 participants	Operating room	Education	Phase 3 team-based skills curriculum for general surgery residents
Malenka et al., 2018	United States	Before and after interventional study	52 handoffs (29 preintervention, 23 postintervention)	Operating room; Intensive Care Unit; Pediatric Intensive Care Unit (PICU)	Protocol	Standardized handoff protocol (including a checklist)
Kinoshita et al., 2009	Japan	Prospective, non-randomised, comparative study	80 participants (47 surgeons and 33 nurses)	Operating room	Education	LADG Basic Lab Course
Zattoni et al., 2017	Italy	Prospective, non-randomised, comparative study	20 simulated emergencies	Operating room	Education and checklist	Surgical team safety training program and institutional checklist
Defontes et al., 2004	United States	Prospective, non-randomised, comparative study	N/A	Operating room	Bundle/Checklists	Preoperative Safety Briefing
Guerlain et al., 2005	United States	Prospective, non-randomised, comparative study	10 surgical cases	Operating room	Audit & Feedback	RATE tool (multitrack, synchronized, digital audio-visual recording system)
Dunn et al., 2007	United States	Before and after interventional study	N/A	Operating room; Intensive Care Unit; Post-anesthesia care unit	Education	Medical Team Training (MTT) program
Marshall et al., 2007	United States	Prospective, non-randomised, comparative study	No specific number provided	Surgical facilities	Education	Human factors program based on Crew Resource Management training
Gururaja et al., 2008	United States	Prospective, non-randomised, comparative study	10 training session videos	Operating room	Education	Debriefing at the Point of Care in Simulation-Based Operating Room Team Training
Edel et al., 2010	United States	Pre-and-post observational study	N/A	Operating room	Bundle/Checklists	Pre-operative Time out/count board
Sewell et al., 2011	UK	Prospective, non-randomised, comparative study	965 surgical cases (480 patients before and 485 patients after the intervention)	Operating room	Education	Educational program (no real name)
Haynes et al., 2011	United States	Pre- and post-intervention survey	257 clinicians actively working in the designated study operating rooms at the eight hospitals participating in a trial of a WHO surgical safety checklist	Operating room	Bundle/Checklists	WHO Surgical Safety Checklist
Forse et al., 2011	United States	Prospective, non-randomised, comparative study	N/A	Operating room	Education	TeamSTEPPS program
Young-Xu et al., 2011	United States	Retrospective health services study	119 383 sampled procedures from 74 Veterans Health Administration facilities that provide care to veterans	Operating room	Education	Veterans Health Administration Medical Team Training (MTT) program
Lee et al., 2012	New Zealand	Before and after interventional study	35,416 procedures; (Phase 1, 10330 procedures) vs (Phase 2, 25086 procedures)	Operating room	Bundle/Checklists	Time Out Procedure (TOP)
Fargen et al., 2013	United States	Before and after interventional study	71 procedures before checklist implementation and 60 procedures after checklist implementation	Operating room	Bundle/Checklists	Neurointerventional-specific WHO surgical checklist
Kawano et al., 2014	Japan	Before and after interventional study	339 responders (177 pre- and 162 post-intervention)	Operating room	Bundle/Checklists	Surgical Safety Checklist (SSCL)
Porter et al., 2014	United States	Before and after interventional study	31 cases	Operating room	Bundle/Checklists	Preprocedural pause (PPP)
Cullati et al., 2014	Switzerland	Cross-sectional study	152 respondents	Operating room	Bundle/Checklists	Surgical Safety Checklist (SSC)
Hsu et al., 2014	Taiwan	Prospective, non-randomised, comparative study	34 staff members	Operating room	Education	Team Resource Management (TRM) Program
Russ et al., 2015	UK	Longitudinal interview study	119 interviews with operating room personnel	Operating room	Bundle/Checklists	World Health Organization (WHO) Surgical Safety Checklist
Hawranek et al., 2015	Poland	Pre-and-post observational study	2,064 cases (1,011 cases pre, 1,053 cases post)	Catheterisation laboratory	Bundle/Checklists	Periprocedural checklist
Hill et al., 2015	UK	Before and after interventional study	113 surgical cases (60 cases at baseline and 53 cases one year later)	Operating room	Bundle/Checklists	The 5 Steps to Safer Surgery (5SSS)
Hsu et al., 2016	United States	Before and after interventional study	103 ICUs	Intensive Care Unit	Education	Comprehensive Unit-based Safety Program (CUSP)
Ong et al., 2015	New Zealand	Pre-and-post observational study	111 operations	Operating room	Bundle/Checklists	"checklist"; adaptation of "WHO Surgical Safety Checklist"
True et al., 2016	United States	Before and after interventional study	108 providers (nursing and medical) in 36 births (18 births per facility)	Perinatal care	Bundle/Checklists	Vaginal Delivery Safety (VaDS) checklist
Chan et al., 2016	China	Cross-sectional study	55 individuals in the departments of Obstetrics and Gynaecology, Anaesthesiology and Operating Theatre Services, Intensive Care Unit and Accident and Emergency	Operating room; Trauma room; Intensive Care Unit	Education	Simulation training using crew resource management
Molina et al., 2016	United States	Before and after interventional study	929 at baseline and 815 at follow-up across 13 hospitals	Operating room	Bundle/Checklists	Surgical Safety Checklist (SSC)
Sucupira et al., 2016	Brazil	Prospective, non-randomized, comparative study	The tool was applied to 486 patients	Operating room	Bundle/Checklists	safety checklist in aesthetic plastic surgery
Riley et al., 2016	United States	Prospective, non-randomised, comparative study	342,754 deliveries	Perinatal care	Audit & Feedback	Premier Perinatal Safety Initiative (PPSI)
New et al., 2016	United States	Before and after interventional study	1120 surgical cases	Operating room	Education	Lean Participative Process Improvement
Riley et al., 2017	United States	Before and after interventional study	26 bed pediatric cardiac ICU	Intensive Care Unit; Cardiac medicine unit	Bundle/Checklists	OR to CICU Handoff
Egenberg et al., 2017	Tanzania	Before and after interventional study	3308 patients	Perinatal care	Education	Scenario-based PPH training
Gillespie et al., 2017	Australia	Pre-and-post observational study	179 surgeries (99 before, 80 after)	Operating room	Education	TEAMANATOMY (brief team training program)
Kuy et al., 2017	United States	Prospective, non-randomised, comparative study	All surgical service staff (88 employees in the surgical service)	Operating room; Perioperative care areas	Education	Crew Resource Management (CRM) training
Leong et al., 2017	Netherlands	Prospective, non-randomised, comparative study	5 surgical teams	Operating room	Bundle/Checklists	Perioperative briefing and debriefing
Gillespie et al., 2017	Australia	Before and after interventional study	520 individual cases (292 pretest, 228 posttest)	Operating room	Education	TEAMANATOMY, a team training program
Lee et al., 2017	United States	Prospective pre-post cohort study	24 surgical cases	Operating room	Education	TeamSTEPPS principles training
Sharma et al., 2018	United States	Non-randomised experimental study	50 cases	Operating room	Protocol	The Whiteboard Technique
Mukhopadhyay et al., 2018	United States	Prospective, non-randomised, comparative study	124 caregivers representing surgery (n=49), anesthesia (n=31) and ICU nursing (n=44)	Operating room; Intensive Care Unit	Bundle/Checklists	The Perioperative Hand Off Protocol
Krimminger et al., 2018	United States	Prospective, non-randomised, comparative study	38 cardiothoracic patients	Intensive Care Unit	Protocol	Standardized handover process and communication template between the OR and the ICU.
Friend et al., 2018	United States	Before and after interventional study	500 video-assisted thoracoscopic surgeries (VATS)	Operating room	Protocol	VATS Kit
Fleetwood et al., 2018	United States	Pre-and-post observational study	4 participants	Simulated Operating Room (SOR)	Simulation	Communication skills training through simulation
Kherad et al., 2018	Canada	Before and after interventional study	2,458 colonoscopies (1,317 colonoscopies at baseline and 1,141 colonoscopies during the intervention period)	Endoscopy unit	Bundle/Checklists	Endoscopy checklist before colonoscopy
Gardezi et al., 2009	Canada	Retrospective claim review	Over 700 surgical procedures	Operating room	Bundle/Checklists	Structured checklist
Seamons et al., 2017	United States	Prospective, non-randomised, comparative study	56 employees	Ophthalmological surgical department	Audit & Feedback	Technology change
Valerio et al., 2017	United States	Pre-and-post observational study	219 participants	Operating room	Bundle/Checklists	Standardized Surgical Checklist (SSC)
Dommaraju et al., 2019	United States	Retrospective study	100 CT-guided procedures	Operating room	Bundle/Checklists	Pre-procedure timeouts
Ravindran et al., 2020	UK	Prospective, non-randomised, comparative study	N/A	Endoscopy units	Cognitive Aid	Endoscopy Team Toolkit
Goldhaber-Fiebert et al., 2020	United States	Prospective, non-randomised, comparative study	69 unique cases	Operating room; Intensive Care Unit; Post-anesthesia care unit	Protocol	The Stanford Emergency Manual - Cognitive Aids for Perioperative Critical Events
Yule et al., 2021	United States	Cross-sectional study	10 surgical experts	Operating room	Education	Gathering Validity Evidence to Adapt the Non-technical Skills for Surgeons (NOTSS) Assessment Tool
Swanson et al., 2021	United States	Quasi-experimental study	50 radiation oncology staff	Radiation oncology department	Education	Crew Resource Management (CRM) Training and Incident Learning System (ILS)
Rose et al., 2018	United States	Prospective, non-randomised, comparative study	54,003 surgical cases	Operating room	Bundle/Checklists	Surgical debriefing checklist
Weldon et al., 2019	UK	Prospective, non-randomised, comparative study	8 surgical simulation courses	Operating room	Education	Video-Supported Simulation of Interactions in the Operating Theatre (ViSIOT)
Zevin et al., 2019	Canada	Prospective cohort study	25 general surgical residents	Operating room	Education	Comprehensive proficiency-based curriculum
Wong et al., 2019	Canada	Pre-and-post observational study	205 interventional radiology procedures	Interventional radiology suite	Bundle/Checklists	Preprocedural checklist
Hemingway et al., 2019	United States	Non-randomised experimental study	56 perioperative nurses	Perioperative environment	Communication device	Hands-free PCD
Randell et al., 2018	UK	Prospective, non-randomised, comparative study	44 theatre staff with experience of robot-assisted colorectal surgery from 9 hospitals	Operating room	Audit & Feedback	Interviews
Geoffrion et al., 2020	United States	Pre-and-post observational study	Video recordings of 64 pre-intervention and 62 post-intervention handoffs	Intensive Care Unit; Cardiac Surgery Intensive Care Unit	Bundle/checklists	Handoff bundle
Lau et al., 2020	United States	Prospective interrupted time series study	22,420 cases (11,447 neurosurgical patients in the preintervention period and 10,973 in the postintervention period)	Operating room	Bundle/Checklists	UC Care Check OR checklist
Soma et al., 2020	Australia	Before and after interventional study	14 patients	Operating room	Bundle/Checklists	Operative team checklist for aerosol generating procedures to minimise exposure of healthcare workers to SARS-CoV-2
Ridley et al., 2021	United States	Prospective, non-randomised, comparative study	141 clinicians (73 surveyed before and 68 after)	Operating room	Education	TeamSTEPPS training
Vortman, 2020	United States	Prospective, non-randomised, comparative study	Not specified (more than 150 employees)	Operating room	Education	Simulation-Based Education for Massive Transfusion Protocol (MTP)
McLaughlin, 2014	United States	Before and after interventional study	93 surgical team members	Operating room	Protocol	Time-out process
Aydin et al., 2021	Turkey	Prospective, non-randomised, comparative study	Interruptions in the operating theatre across 52 surgical procedures (12 neurosurgery and 40 general surgery operations) were recorded. Operations were performed by seven surgical teams at two tertiary care centres. routine operative procedures (ROP, n=26, observed without any intervention) and intervened operative procedures (IOP, n=26, observed after implementation of preventive measures)	Operating room	Bundle/Checklists	Intervened operative procedures
Towning et al., 2021	UK	Prospective, non-randomized comparative study	94 staff participants	Operating room	Education	Simulation Training for Surgical Tracheostomy
Chilakapati et al., 2021	United States	Pre-and-post observational study	The pre- and post-implementation surveys were completed by 19 and 26 operating room staff members, respectively	Operating room	Cognitive aid	Strabismus-specific whiteboard
Ber et al., 2021	United States	Before and after interventional study	637 and 893 cases during the preintervention and intervention periods, respectively.	Operating room	Protocol	Aviation-like structured team communication practices
Urban et al., 2021	Canada	Cross-sectional study	2,032 healthcare professionals	Operating room	Bundle/Checklists	Surgical Safety Checklist
Sujan et al., 2022	UK	Before and after interventional study	14 participants	Surgical Emergency Unit	Education	Functional Resonance Analysis Method (FRAM)
Sillero et al., 2021	Spain	Prospective, non-randomised, comparative study	16 surgical staff	Operating room	Audit & Feedback	In-depth interviews
Truong et al., 2021	United States	Two-wave survey study	208 participants	Operating room	Education	Multidisciplinary simulated operating room (OR) team training
Grogan et al., 2022	United States	Cross-sectional study	72 participants	Operating room	Protocol	Identifier Bouffants
Van Dalen et al., 2022	Netherlands	Prospective, non-randomised, comparative study	98 study participants	Operating room	Protocol	Theatre cap challenge
Kalantari et al., 2021	Iran	Randomised controlled trial	300 nurses	Operating room	Education	Intraoperative education session
Vigo et al., 2022	Switzerland	Prospective, non-randomised, comparative study	175 robotic laparoscopic procedures	Operating room	Education	Interdisciplinary surgical team-training protocol for robotic gynecologic surgery
Hartman et al., 2022	United States	Pre-and-post observational study	3 liver transplant teams	Operating room	Education	Veno-veno bypass simulation
Thomas et al., 2019	United States	Prospective, non-randomised, comparative study	960 surgical phrases (480 spoken via the Da Vinci Si speakers, and 480 expressed through a wireless, hands-free system)	Operating room	Other	Wireless, hands-free audio system
Guris et al., 2019	United States	Prospective observational cohort study with a double-blind and randomised controlled component	22 1st-year anaesthesiology residents	Operating room	Education	Simulation education +/- a didactic session on speaking up behaviour
Acar et al., 2019	Turkey	Prospective, randomized simulation study	A total of 19 scenarios were run with 28 participants	Operating room	Bundle/Checklists	Standardized evacuation checklist
Urman et al., 2021	United States	Randomized controlled trial	304 anesthesiologists (95 simulations)	Post-anesthesia care unit; Emergency Department	Education	Emergency manual (EM)
Sharma et al., 2021	Canada	Prospective Cohort Study	144 laparoscopic operations	Operating room	Protocol	Device-related interruptions characterized using the OR Black Box
Turrentine et al., 2020	United States	Prospective, non-randomised, comparative study	208 medical students (67 postintervention and 141 preintervention)	Operating room	Education	Simulated Room of Errors
Soares et al., 2021	Brazil	Methodological Study	24 participants	Simulation Laboratory of Clinical Practice in Nursing and Health	Education	Professional Nursing Communication Competence (IMC- CPE)
Tsafrir et al., 2020	United States	Non-randomized, prospective controlled trial	137 procedures	Operating room	Other	Quail Digital Healthcare headset system
Hussain et al., 2020	Pakistan	Qualitative case study research	16 health professionals	Operating room; Perinatal care	Education	Multidisciplinary team training
Suresh et al., 2021	India	Cross-sectional study	200 cases	Operating room	Bundle/Checklists	Modified WHO SSC for Neurosurgery
Shi et al., 2021	United States	Before and after interventional study	22 surgical staff	Operating room	Education	In-situ simulations
Wai et al., 2021	Hong Kong	Pre-and-post observational study	46 students	Classroom	Education	Crew Resource Management Training
Catchpole et al., 2022	United States	Pre-and-post observational study	367 trauma cases	Trauma room	Cognitive aid	“in the wild” smartphone communication app
Douglas et al., 2021	Australia	Before and after interventional study	107 operating room staff members	Operating room	Protocol	Surgical caps displaying team members’ names and roles
Nasiri et al., 2021	Iran	Prospective, non-randomised, comparative study	120 handovers	Operating room	Bundle/Checklists	SWITCH Checklist
